# Unlocking the Potential of ctDNA in Sarcomas: A Review of Recent Advances

**DOI:** 10.3390/cancers17061040

**Published:** 2025-03-20

**Authors:** Sahana Aiyer, Tae-Hee Kim, Katharine Collier, Raphael Pollock, Claire Verschraegen, Daniel G. Stover, Gabriel Tinoco

**Affiliations:** 1 College of Medicine, The Ohio State University, Columbus, OH 43210, USA; sahana.aiyer@osumc.edu (S.A.); tae-hee.kim@osumc.edu (T.-H.K.); 2 Division of Medical Oncology, Department of Internal Medicine, The Ohio State University Comprehensive Cancer Center, Columbus, OH 43210, USA; katharine.collier@osumc.edu (K.C.); claire.verschraegen@osumc.edu (C.V.); daniel.stover@osumc.edu (D.G.S.); 3 Department of Surgery, The Ohio State University Comprehensive Cancer Center, Columbus, OH 43210, USA; raphael.pollock@osumc.edu

**Keywords:** soft tissue sarcoma, ctCNA, digital droplet PCR, ddPCR, next-generation sequencing, NGS, whole-genome sequencing, WGS

## Abstract

Soft tissue sarcomas (STSs) constitute a group of tumors with heterogeneous alterations and different biological behavior. Genetic profiling techniques have immense potential to revolutionize sarcoma classification, detection, and treatment. Cell-free DNA (cfDNA) analysis offers a minimally invasive approach to profiling tumor alterations, including tracking specific mutations or targeted panels of cancer-related genes via DNA sequencing methods. Circulating tumor DNA (ctDNA) platforms have gained popularity as a noninvasive alternative to tissue biopsies, offering a less invasive approach to tumor profiling. Nonetheless, ctDNA profiling in concordance with standard solid tumor comprehensive genomic profiling (CGP) is poorly characterized for STSs. Ultra-low-pass whole-genome sequencing and whole exome sequencing of cfDNA have yet to be fully leveraged in patients with sarcomas. This comprehensive review provides an overview of the application of ctDNA in STSs.

## Simple Summary

Soft tissue sarcomas (STSs) are rare, heterogeneous malignancies with complex genetic profiles, presenting significant diagnostic and therapeutic challenges. Despite advances in treatment, the outcomes of metastatic STS remain poor, highlighting the need for novel biomarkers. Circulating tumor DNA (ctDNA) has emerged as a promising noninvasive tool for disease monitoring, prognosis, and treatment response. Advances in detection methods, including PCR, next-generation sequencing, and low-pass whole-genome sequencing, have enabled the identification of sarcoma-specific mutations and structural variants. Studies have demonstrated correlations between ctDNA levels and tumor burden, treatment efficacy, and recurrence. While challenges remain, ongoing research underscores ctDNA’s potential to revolutionize sarcoma management and personalized care.

## 1. Background

### 1.1. Sarcoma Introduction

Soft tissue sarcomas (STSs) are rare malignancies of mesenchymal origin, accounting for approximately 1% of adult cancers, with an estimated 13,500 new cases diagnosed in 2020 in the United States [1]. STSs constitute a heterogeneous and complex group of tumors with around 100 different histologic and molecular subtypes and variable clinical behavior [2]. An estimated 50% of patients with metastatic STS die within 18 months of their initial diagnosis [1,2].

Based on their genomic characteristics, STSs can be classified into two groups [3]. The first is characterized by near-diploid genomes and relatively simple genetic alterations, including oncogene-activating translocations. This group constitutes around one-third of all STS cases overall [3]. The second group is characterized by high levels of genomic instability. These sarcomas present an extensive range of abnormalities, such as somatic copy number alterations (SCNAs) [4], including chromosome copy number changes, unbalanced translocations, amplifications, deletions, and chromothripsis. Most STSs are in this group [3,5].

Sarcomas with complex genomic profiles include undifferentiated pleomorphic sarcomas, leiomyosarcomas (LMSs), malignant peripheral nerve sheath tumors (MPNSTs), dedifferentiated liposarcomas, and pleomorphic liposarcomas, accounting for nearly 50% of sarcoma cases [4,6]. These cytogenetically complex sarcomas are often aggressive, putting patients at high risk of developing locally advanced or metastatic disease [4,6].

Wide excision plus radiotherapy remains the cornerstone treatment for non-metastatic tumors and is considered the standard of care. The role of perioperative chemotherapy in this setting is limited to specific high-risk sarcomas [7,8,9]. The treatment of advanced sarcomas remains a major therapeutic challenge. Historically, the mainstay treatment has been conventional chemotherapy; however, recently, targeted therapies have become more relevant in the field [10,11]. There are no data to support the optimal management of patients presenting with metastatic disease. The therapeutic modality—including systemic therapies, surgery, and radiation therapy—must be determined on a case-by-case basis, considering a tumor board’s recommendations when possible [10,11]. Thus, there is a critical need to develop reliable biomarkers for prognosis, systemic treatment selection, treatment monitoring, and early detection of disease progression and relapse [12].

### 1.2. cfDNA/ctDNA Introduction

Liquid biopsy (LB) is a term used to describe the detection of circulating tumoral elements, which include circulating tumor cells (CTCs), circulating tumor DNA (ctDNA), circulating tumor RNA (ctRNA), tumor-educated platelets, and exosomes [13].

ctDNA has emerged as a novel approach for identifying oncogenic mutations, measuring disease burden, carrying out clinical prognostication, and assessing tumor response to therapy [14,15,16].

**Figure 1:**
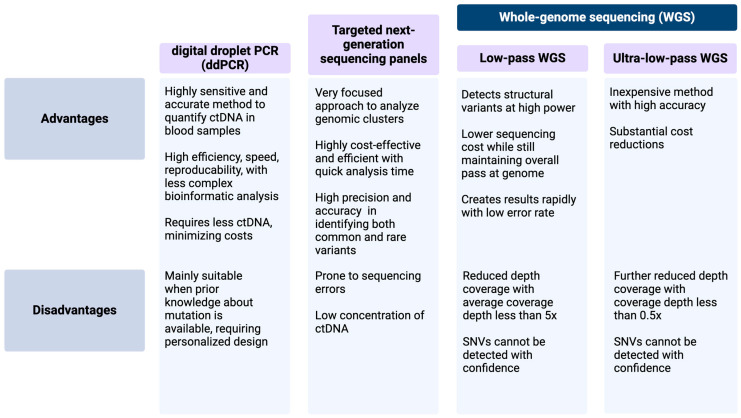
Techniques utilized to detect ctDNA in sarcomas, designed in Biorender [30,31,32,33,34].

This paper will mainly discuss the technologies available for ctDNA detection and relevant studies on sarcoma (See Figure 1 for the techniques utilized to detect ctDNA in sarcomas and Table 1 for a list of current ctDNA trials in sarcoma).

Since the initial detection of tumor-derived DNA fragments in 1948 [17], novel technological advances have made it possible to better characterize and analyze ctDNA [18,19,20]. However, the use of ctDNA technologies in sarcomas is very recent and still in the early phases of development.

Cell-free DNA (cfDNA) is defined as extracellular nucleic acids that can be found in body fluids, such as plasma, urine, and cerebrospinal fluid [21]. cfDNA primarily consists of normal DNA from leukocytes and other normal cells; thus, it can be detected in patients without malignancy [22,23,24,25,26]. A smaller fraction of cfDNA, known as ctDNA, is derived from tumors in patients with cancer [27,28,29].

**Table 1:** Studies on ctDNA in sarcomas.

Authors	Technique	Sarcoma Type(s)	Sensitivity/ Specificity	Correlation with Clinical Outcomes	Other Critical Findings
Schmidkonz and Krumbholz et al. [35]	18F-FDG-PET/CT + ctDNA quantification (ddPCR)	ES	PET-ctDNA agreement: 65% after 2 cycles, 90% after 5 cycles	Correlation between PET-derived parameters and ctDNA; effective for response monitoring and relapse detection	Combined PET/ctDNA offers promising noninvasive monitoring
Shah and Azad et al. [36]	CAPP-Seq and CNA detection	ES, RMS, OS	Translocations identified with 81.3% sensitivity	Declines in ctDNA levels aligned with therapy response	Rising ctDNA detected before relapse, providing early monitoring potential
Ruhen and Lak et al. [32]	ctDNA via ddPCR, panel sequencing, WES	Pediatric RMS	ctDNA detected: 14/18 pretreatment plasma (ddPCR), 7/7 sequencing	Fluctuations in ctDNA corresponded to treatment response	Sequencing better for tracking changes across multiple genomic targets
Lyskjaer and Davies et al. [37]	ddPCR	Chondrosarcoma	Pre-op ctDNA: 37%; post-op ctDNA: 39%	Detection correlated with poor prognosis, tumor grade, and volume	Hotspot SNVs detected in 80% of cases
Christodoulou and Yellapantula et al. [38]	LB with low-pass WGS	Pediatric solid tumors: sarcomas, renal tumors, hepatic tumors	CNA detection rate ~70%	Feasible for stratifying pediatric patients and early relapse detection	Hybridization-based panel successfully identified sarcoma-related fusions
Ruas and Silva et al. [39]	LB-based CNA profiling (ddPCR)	Pediatric tumors: ES, RMS, LMS	CNA levels concordant between tumors and ctDNA: 56%	cfDNA levels correlated with metastasis development during therapy	Potential for long-term biomarker monitoring
Audinot and Drubay et al. [40]	Low-pass WGS	OS	Improved clinical score discrimination by >8% and ~5% for different time points	Prognostic factor for OS risk stratification when combined with known clinical factors	PRONOS tool improves prognosis predictions with diagCPA
Lyskjaer and Kara et al. [41]	Methylation-specific ddPCR	OS	ctDNA detected in 69% of samples pre-surgery	Effective for prognosis and survival prediction	Pre-surgical levels correlated with survival outcomes and prognosis
Bui and Nemat-Gorgani et al. [42]	Patient-specific ctDNA analysis using bespoke panels	STS	ctDNA increased in 90% of patients post-cryotherapy	Subsequent decreases in ctDNA correlated with better PFS	Pretreatment ctDNA allele fraction negatively associated with treatment response and overall survival
Anderson and Ghisoli et al. [43]	Patient-specific ddPCR	Recurrent ES	Patient-specific EWS/FLI1 ctDNA detected in all 8 patients at baseline	ctDNA levels tracked disease burden during treatment	Correlated with treatment response to Vigil/TEM/IRI
Mattox and Douville et al. [44]	RealSeq and ddPCR	MPNST	Sensitivity: 33% (aneuploidy), 50% (CNA) with specificity: 97%	LB effective for early detection of MPNST	High specificity retained with sub-chromosomal CNA analysis
McConnell and Gazdova et al. [45]	Sarcoma-specific NGS gene panel	ARMS, ES, synovial sarcoma, extraskeletal myxoid chondrosarcoma, clear cell sarcoma, undifferentiated round cell sarcoma, myxoid liposarcoma, alveolar soft part sarcoma, dedifferentiated liposarcoma	Variants identified: 91.6% tissue, 50% plasma	NGS in ctDNA shows great potential for diagnosis and monitoring of various sarcomas	N/A
Lak and van Zogchel et al. [32]	S-WGS and cfRRBS	Pediatric RMS	ctDNA detection: 53% and 92% (cfRRBS)	Plasma RASSF1A correlated with poor prognosis	Methylation-specific detection methods proved effective for risk stratification
Demoret and Gregg et al. [46]	Targeted NGS	LMS, GIST, dedifferentiated liposarcoma, undifferentiated pleomorphic sarcoma	ctDNA detection was feasible in 77% of cases, with moderate overall concordance rate of 51%	Partial correlation with clinical outcomes	26% complete concordance, 46% partial concordance, >50% TF < 1%
Heinrich and Jones et al. [47]	Targeted NGS	Advanced GIST	ctDNA detected in 77%; KIT mutations: 59%	KIT exon 11 mutations associated with improved PFS	ctDNA sequencing predicts therapy efficacy
Johansson and Berndsen et al. [48]	Targeted NGS	Advanced GIST	ctDNA detected in at least 1 time point: 28%	High-risk patients exhibited higher ctDNA levels and tumor cell proliferation	Patients positive for ctDNA pre-surgery became ctDNA-negative post-surgery
Braig and Runkel et al. [49]	Targeted NGS	STS	Mean conversion rate: 51.4%	Statistically significant correlation with tumor burden and treatment response	ctDNA levels indicated minimal residual disease during remission and increased with recurrence
Zhou and Bui et al. [50]	Targeted NGS	LMS	100% ctDNA positivity concordant with imaging-detected disease progression	ctDNA levels concordant with disease progression and therapy response	Key driver mutations included TP53, PTEN, and RB1
Eisenhardt and Schmid et al. [51]	Targeted NGS	Myxoid liposarcoma	Breakpoints of balanced translocations detected in 70.5% of tumors	ctDNA levels decreased post-resection and increased with metastatic disease	ctDNA declined with radiotherapy/chemotherapy but rose with increased tumor burden
Eisenhardt and Brugger et al. [52]	Targeted NGS	Synovial sarcoma	ctDNA breakpoint detection: 100% specificity, 40% sensitivity	Increased ctDNA mutations improved sensitivity and correlated with tumor burden	Exome panel proved more sensitive and valuable than breakpoint panel
Madanat-Harjuoja and Klega et al. [53]	ULP-WGS	LMS	ctDNA detection: 49% pre-chemo, 24.6% post-2 cycles of chemo	Higher ctDNA correlated with lower survival; post-2 cycles of chemo linked to worse outcomes	N/A
Szymanski and Sundby et al. [54]	ULP-WGS	MPNST	TF distinguished MPSNT from PN with 86% accuracy	cfDNA distinguished benign vs. malignant tumors in hereditary cancer syndrome	ctDNA levels correlated with radiographic response across treatments
Abbou and Klega et al. [33]	ULP-WGS + Rhabdo-Seq	IR-RMS	Rhabdo-Seq detected ctDNA in 55% of cases	Higher ctDNA levels were linked to significantly worse outcomes	PAX3/PAX7-FOXO1 translocations detected with precision
Tsoi and Gokgoz et al. [55]	WES + ddPCR analysis	Bone and soft tissue sarcoma	cfDNA detected in 69/70 samples (>0.5 ng/mL)	Lower cfDNA levels were associated with improved disease-free survival	N/A
Seidel and Kashofer et al. [31]	WGS and ddPCR	ES	N/A	ctDNA detection correlated with treatment response and remission; potential for monitoring and stratification	Strong indicator of disease recurrence

Abbreviations: ARMS—alveolar rhabdomyosarcoma; CNA—copy number alteration; cfRRBS—cell-free reduced representation bisulfite sequencing; ctDNA—circulating tumor DNA; ddPCR—digital droplet polymerase chain reaction; diagCPA—ctDNA quantification at diagnosis; ES—Ewing sarcoma; GIST—gastrointestinal stromal tumor; LB—liquid biopsy; LMS—leiomyosarcoma; MPNST—malignant peripheral nerve sheath tumor; NGS—next-generation sequencing; OS—osteosarcoma; PFS—progression-free survival; RMS—rhabdomyosarcoma; SNV—single-nucleotide variant; STS—soft tissue sarcoma; S-WGS—shallow whole-genome sequencing; TF—tumor fraction; ULP-WGS—ultra-low-pass whole-genome sequencing; WES—whole-exome sequencing; WGS—whole-genome sequencing.

## 2. DNA Profiling and Detection Methods

### 2.1. Commonly Used Methods

To detect and quantify ctDNA in cancer patients, profiling techniques optimized to identify somatic hallmarks that differentiate tumor DNA from germline DNA are required. In cancer, somatic variants are generally categorized as structural variants that include copy number variants (CNVs) and rearrangements, single-nucleotide variants (SNVs), and epigenomic alterations, including DNA methylation and histone modifications.

Polymerase chain reaction (PCR) and next-generation sequencing (NGS) [19,56] are the two main laboratory techniques used to study somatic events of interest, such as SNVs, CNVs, translocations, and DNA methylation. The development of digital technologies has greatly improved PCR assays’ sensitivity and quantitative ability to detect somatic DNA variants. Still, the PCR approach requires pretest knowledge of the genomic regions or specific variants of interest [12,19]. PCR techniques are well suited for genotyping tumors in cancers driven by recurrent hotspot mutations, particularly SNVs. Still, as most sarcomas are caused by structural variants or loss-of-function variants with interpatient variability in the genomic location, PCR approaches are required to be multiplexed or designed for individual patients [12,57].

NGS approaches for detecting ctDNA are now used more commonly due to rapidly dwindling sequencing costs and improved sensitivity of the techniques. NGS can profile the entire genome for different sets of genes or specific genomic locations [56,58]. The vast majority of ctDNA studies in sarcoma have focused on the detection of SNVs, structural variants, DNA methylation, and a few histone modifications in ctDNA.

Emerging data have revealed the potential use of ctDNA in a few sarcoma types [59,60,61,62,63,64,65]. As sarcomas shed low levels of cfDNA that limit ctDNA identification, novel, consistent, cost-effective methods for ctDNA detection are needed.

### 2.2. ctDNA Detection Technologies Used in Sarcoma

The genomic landscape of the most common sarcomas has been recently described [66,67,68,69,70]; as more data have become available, cfDNA analysis has focused primarily on tumor-specific genetic aberrations, including chromosomal translocations (fusion genes) and copy number alterations (CNAs) [71].

The most commonly used assays include digital droplet PCR (ddPCR), targeted NGS panels, exome sequencing, and low-coverage whole-genome sequencing (WGS) [35,56,60,72,73,74,75]. However, to use these technologies, prior knowledge of chromosomal breakpoints (ddPCR) and focusing on only one or a few genetic alterations (ddPCR or targeted NGS panels) are required [71]; moreover, these technologies may suffer from low sensitivity [76,77,78].

The SNVs commonly found in sarcomas are characterized by loss-of-function mutations of tumor suppressor genes and recurrent patterns of structural variants, including CNAs and translocations. In contrast to carcinomas, recurrent gain-of-function SNVs are rare in sarcomas [12]. Other important somatic findings in sarcomas include aneuploidy [59] and alteration in genome-wide methylation patterns [66].

WGS has been applied to study ctDNA in sarcomas. More recently, new platforms have developed low-coverage WGS to detect the presence of CNAs and quantify the fraction of the sample containing ctDNA [59,60,65].

Newer targeted NGS techniques, such as ultra-low-pass WGS (ULP-WGS) and whole exome sequencing of ctDNA (ctWES), are useful in detecting SCNAs in ctDNA and provide a measure of the tumor fraction (TF) of ctDNA present in blood samples [58,59]. Currently, efforts to explore its use in sarcoma are ongoing.

#### 2.2.1. ddPCR Analysis

ddPCR analysis is a powerful molecular technique that quantifies ctDNA in blood samples [30]. It is a highly sensitive and accurate method that is being increasingly used to monitor patients with cancer and manage their treatment due to its reproducibility and efficiency. ddPCR is a fast and sensitive method for detecting ctDNA mutations. Various mutations can be analyzed simultaneously in one reaction, minimizing the amount of ctDNA required as well as costs [30].

Mattox and Douville et al. employed ddPCR analysis with RealSeQ to better understand ctDNA in MPNST to differentiate from benign neurofibroma [44]. Their goal was to calculate genome-wide aneuploidy scores (GAS) to detect MPNST. Using this RealSeQ and ddPCR analysis, they found an overall sensitivity of 33% for MPNST detection and an improvement of 50% when analyzing sub-chromosomal CNAs while retaining a high specificity of 97%. ddPCR was able to identify mutations in NF1, NF2, RB1, TP54BP2, and GOLGA2. Based on these findings, ddPCR LB shows great promise in identifying early-stage MPNST [44]. ctDNA may be a valuable biomarker of MPNST progression.

Ruas and Silva reported a study evaluating CNAs in ctDNA as a biomarker for monitoring pediatric cancer, identifying recurrent alterations in MYCN (42.9%), 1q (35.7%), and 17p (28.6%) across neuroblastoma, Wilms tumor, and sarcomas. cfDNA levels were significantly higher in neuroblastoma (median 427.5 ng/mL) compared to Wilms tumor (median 99.4 ng/mL) and sarcomas (median 20.3 ng/mL). A high concordance (83%) between tumor and ctDNA was observed, though 17.2% of CNAs were exclusively detected in cfDNA, reflecting tumor heterogeneity [39].

In addition to ctDNA detection, ddPCR analysis has also been shown to predict tumor burden and treatment response in various sarcoma tumors. Many studies have utilized ddPCR analysis to associate ctDNA levels with clinical outcomes, creating a potentially valuable biomarker for future routine monitoring in the healthcare setting.

Anderson and Ghisoli et al. evaluated the safety and efficacy of the Vigil treatment for Ewing sarcoma (ES) patients [43]. They performed patient-specific ddPCR to quantify ctDNA and identify EWS/FLI fusion gene translocation breakpoints and found a statistically significant correlation between changes in ctDNA levels and changes in disease burden throughout treatment. After the initiation of Vigil therapy, ctDNA levels dropped below detection levels but increased again after therapy discontinuation. These results demonstrate the value of ddPCR ctDNA analysis in monitoring treatment response and disease recurrence, providing a tool that could be employed in routine clinical tests [43].

Similarly, Ruhen and Lak et al. investigated ctDNA quantification as a marker of disease burden and/or treatment response in pediatric rhabdomyosarcoma (RMS) samples [79]. They applied ddPCR to detect ctDNA and tumor-specific genetic variants. They found that fluctuations in ctDNA levels statistically significantly correlated with treatment response. ctDNA levels at diagnosis were significantly higher in those with unfavorable tumor sites, positive nodal status, and metastasis. These findings highlight how ctDNA detection through ddPCR analysis is a potential minimally invasive biomarker [79]. Prospective clinical trials are still needed to apply these monitoring techniques in the clinical setting to predict tumor response and progression.

Another study by Tsoi and Gokgoz et al. explored the prognostic significance of ctDNA in bone and soft tissue sarcomas utilizing ddPCR [55]. They reported statistically significantly improved disease-free survival in patients with lower ctDNA levels. This shows the potential clinical utility of ctDNA as a biomarker for bone and soft tissue sarcoma disease monitoring and stratification [55].

Lyskjaer and Davies et al. investigated ctDNA-level correlations with health outcomes in chondrosarcoma patients using a ddPCR system [37]. Interestingly, they found that ctDNA detection was more accurate than pathology in identifying high-grade tumors. Patients with higher levels of ctDNA had a statistically significantly poorer prognosis and risk of relapse. Pre-operation ctDNA detection corresponded with tumor grade, and failure to detect ctDNA pre-operation correlated with a good prognosis and well-differentiated tumors. These authors also reported ctDNA to be statistically significantly associated with tumor volume; in addition, ctDNA detection pre-operation was related to increased tumor size. These results hold great promise but are only based on a small sample size of patients. Before this blood test can be used as a routine and complementary biomarker in clinical practice, larger validating studies are needed [37].

Similarly, Lyskjaer and Kara et al. employed methylation-specific ddPCR assays to identify ctDNA in osteosarcoma (OS) patients [41]. Researchers analyzed over 11,500 samples, including tumor, blood, and plasma data, to identify and validate four key methylation markers. ctDNA was detected in 69% of preoperative plasma samples using a single-marker approach and in 40% of samples when applying a dual-marker threshold. These findings correlate with disease progression and overall survival, highlighting ctDNA as a promising noninvasive tool for monitoring disease, stratifying risk, and assessing treatment response in OS. Larger studies are needed to confirm clinical utility. These findings highlight how methylation-specific ddPCR assays could be effective in predicting prognosis and risk stratification in patients with OS, allowing for the potential application of routine clinical biomarkers and monitoring [41].

Just like other ctDNA analysis methods, ddPCR offers a promising approach to link ctDNA with sarcoma disease outcomes and treatment responses. ddPCR can be performed across various sarcoma tumors, with all studies highlighting its efficacy in correlating ctDNA with tumor burden. Similarly to the previously mentioned analysis techniques, larger prospective studies must be implemented to validate these results before such assays can be applied in the clinical setting. Regardless, ddPCR shows great potential in detecting ctDNA as a clinical biomarker for predicting prognosis and aiding in treatment decisions.

#### 2.2.2. Whole-Genome Sequencing

WGS is the comprehensive sequencing of the whole genome, including coding and noncoding sequences [80]. WGS analyzes both small and large variants that more targeted strategies could miss. Additionally, it is beneficial in capturing a large amount of data rapidly and tests for a broad range of variant types simultaneously [80].

Seidel and Kashofer et al. investigated the feasibility of ctDNA detection through WGS in pediatric ES to improve disease monitoring and treatment stratification [31]. They performed WGS to detect tumor breakpoints in tumors containing the EWS-FL1 fusion gene. These breakpoints were detected in blood plasma through ddPCR before, during, and after treatment. They found that the presence and levels of fusion breakpoints were statistically significantly associated with ES clinical outcomes. They reported a positive correlation between molecular ctDNA response, treatment response, and ongoing remission. Increasing ctDNA levels were shown to exhibit a strong indication of ES disease recurrence, treatment failure, and disease advancement. Seidel and Kashofer et al.’s findings allow for the incorporation of ctDNA data as a complementary clinical tool in the routine monitoring of patients with ES [31].

Although ctDNA levels were statistically significantly correlated with ES clinical outcomes, there was high variability between patients. Thus, study validation and replication with a larger sample size and randomized trial implementation are important.

#### 2.2.3. Low-Pass and Ultra-Low-Pass WGS

Low-pass WGS (LP-WGS) is a high-throughput DNA sequencing technique utilized to detect structural variants at higher statistical power and map their breakpoints [81]. Each base in the selected genome is sequenced a few times, producing reduced depth coverage. The average coverage depth is less than 5X but can be as low as 0.1X. While SNVs cannot be determined with confidence using LP-WGS, the advantage of reduced depth coverage is a lower sequencing cost while still achieving a generalized pass at the whole genome. LP-WGS requires low-input DNA, generates results rapidly, and yields a low error rate. LP-WGS has been explored for its role of detecting and quantifying ctDNA to stratify sarcoma patients [81].

Christodoulou and Yellapantula et al. designed an LB system with LP-WGS to identify CNAs and mutations in pediatric solid tumors consisting of STSs, renal tumors, germ cell tumors, hepatic tumors, and thyroid tumors [38]. The cfDNA was analyzed from plasma acquired before, during, and after therapy, as well as at relapse. CNAs were identified in 53% of patients enrolled at relapse and 80% of patients with metastatic disease; these CNAs successfully matched the CNAs detected by chromosomal microarray analysis in the same tumors. The overall CNA detection rate obtained using LP-WGS was approximately 70%, indicating clinical feasibility in monitoring and stratifying pediatric patients with a variety of solid tumors. As shown by the 70% sensitivity in LP-WGS, carrying out serial LB of patients is a sensitive method for detecting early response and relapse. Additionally, these authors established a hybridization-based panel that successfully identified *EWSR1* and *FOX01* fusion genes in 10/12 ES plasma samples and 2/2 alveolar RMS (ARMS) plasma samples. These findings highlight the effectiveness and potential of LP-WGS in tracking and stratifying pediatric sarcoma patients [38].

Similarly, Audinot and Drubay et al. reported success in ctDNA quantification and the monitoring of OS samples using LP-WGS [40]. They utilized data from the OS2006 prospective protocol to determine the effectiveness of a novel ctDNA quantification method in predicting high-grade OS health outcomes. LP-WGS was performed to sequence plasma from patients at OS diagnosis time, before surgery, and at the end of treatment. ctDNA quantification at diagnosis (diagCPA) was determined as a major prognostic factor for progression-free survival and overall survival, independent of other clinical factors such as metastasis identification and histological response. When adding diagCPA to metastasis identification, OS patient outcome stratification and prediction improved. diagCPA improved clinical score discrimination by >8% and ~5% for different time points. Using these findings, Audinot and Drubay et al. established a prediction tool, PRONOS, that developed individual risk predictions for patients with OS, demonstrating effective ctDNA quantification at surgery and after treatment [40].

Lak and van Zogchel et al. utilized shallow WGS (sWGS), also known as LP-WGS, to investigate the diagnostic potential of cfDNA and ctDNA in pediatric RMS, yet another type of sarcoma [32]. This study aimed to correlate methylated *RASSF1A*, a tumor suppressor gene commonly silenced by methylation in adult and pediatric tumors, with RMS health outcomes and survival analysis. Using advanced molecular techniques, including sWGS, reduced representation bisulfite sequencing (cfRRBS), and ddPCR for RASSF1A methylation (RASSF1A-M), the researchers demonstrated high feasibility for detecting tumor-derived DNA. RASSF1A-M positivity correlated significantly with poor outcomes, especially in metastatic cases, with a 5-year event-free survival rate of 46.2% compared to 84.9% for RASSF1A-M-negative cases. This demonstrates the strong prognostic potential of *RASSF1A-M* detection in cfDNA and its clinical potential in improving prognosis prediction and risk stratification in RMS [32].

These three studies used LP-WGS to successfully monitor and track patients with distinct forms of sarcoma. Prediction tools that incorporate LP-WGS ctDNA analysis, such as PRONOS and RASSF1A-M detection, pose great potential in predicting survival, early response to treatment, relapse, and other clinical risk factors. Applying these tools to larger cohorts of patients is required to validate their clinical applicability in monitoring and predicting patients’ sarcoma disease status.

Ultra-low-pass WGS (ULP-WGS) has recently emerged as an inexpensive tool to accurately detect both ctDNA and large structural genomic variations [82]. Compared to WGS and LP-WGS, it provides reduced sequencing coverage of <0.5X but with substantial cost reductions, making it a promising alternative [82].

Madanat-Harjuoja and Klega et al. investigated whether ctDNA detection in patients undergoing chemotherapy for advanced LMS is associated with survival [53]. The scientists performed ULP-WGS to detect ctDNA and reported that ctDNA was detectable in 49% of advanced LMS patients prior to chemotherapy treatment and in 24.6% of patients after two cycles of treatment. A higher ctDNA rate was statistically significantly associated with a lower survival rate, and patients with detectable rates of ctDNA after two cycles of chemotherapy demonstrated significantly worse overall survival. Interestingly, the detection of copy number gains of *MYC* and loss of *PTEN* was correlated with worse overall survival and decreased progression-free survival. The takeaway that ctDNA detection through ULP-WGS is associated with patient outcome and chemotherapy response in advanced LMS patients raises important implications for the care of patients with advanced or metastatic LMS. Additionally, the detection of a higher ctDNA level before chemotherapy indicates that the patient is less likely to benefit from chemotherapy, information that is essential for treatment decisions and stratification [53]. These findings highlight the potential of ctDNA inclusion as an integral tool in the routine monitoring of patients with LMS.

Szymanski and Sundby et al. also analyzed ctDNA levels through ULP-WGS to better understand the transformation of benign plexiform neurofibroma (PN) to MPNST [54]. They found that cfDNA from MPNST patients demonstrates a shorter fragmentation profile compared to PN patients or healthy donors. They reported that TF in plasma cfDNA distinguished pretreatment MPSNT from PN with 86% accuracy. Interestingly, cfDNA from MPNST patients displayed statistically significantly increased tumor genomic instability compared to PN cfDNA. Plasma-derived TF was also significantly correlated with tumor size from imaging performed in MPNST patients. These cfDNA analyses exhibit the potential for the noninvasive detection of minimal residual disease and treatment response assessment. Szymanski and Sundby et al.’s cfDNA fragment analysis with ULP-WGS is novel in distinguishing between benign and malignant tumors in a heritable cancer predisposition syndrome [54]. This cfDNA analysis holds significant potential in biomarker development for treatment response and as a screening assay for the early detection of MPNST. These findings are valuable in guiding personal treatment escalation since they provide more knowledge on when a premalignant lesion transforms into cancer.

Interestingly, ULP-WGS has been used to stratify risk in yet another form of sarcoma. Abbou and Klega et al. utilized ULP-WGS to detect ctDNA in intermediate-risk RMS (IR-RMS); they investigated whether ctDNA detection pre-therapy is correlated with health outcomes [33]. They also devised a new custom RMS-specific sequencing assay (Rhabdo-Seq) to identify rearrangements and SNVs. They were able to differentiate between fusion-negative RMS (FN-RMS) and fusion-positive RMS (FP-RMS). Abbou and Klega et al. found that IR-RMS patients with detectable ctDNA at diagnosis exhibited statistically significantly worse clinical outcomes than those without detectable ctDNA. However, through multivariable analysis, ctDNA was independently associated with worsened clinical prognosis in the FN-RMS group but not in the smaller FP-RMS group. Rhabdo-Seq was able to detect pathognomonic translocations between PAX3 or PAX7 and FOXO1 and was shown to measure ctDNA very effectively, a significant takeaway. Specifically, Rhabdo-Seq was able to detect ctDNA in 55% of FN-RMS cases. Abbou and Klega et al.’s study highlights how baseline ctDNA detection through ULP-WGS is possible and prognostic in IR-RMS [33].

Similarly to LP-WGS, all three studies investigated the use of ULP-WGS in ctDNA detection and sarcoma risk prediction. They analyzed different types of sarcomas and found ctDNA analysis to provide valuable prognostic and disease monitoring potential. ctDNA levels measured through ULP-WGS statistically significantly correlated with clinical outcomes in all three studies, emphasizing the need for ctDNA analysis in routine disease monitoring. There is always room for improvement with ULP-WGS sensitivity, and larger prospective studies with further validation techniques are required to make ctDNA a staple measure of risk-stratified treatment strategies and disease prediction.

#### 2.2.4. Targeted NGS

Targeted NGS focuses on a specific cluster of genomic regions to analyze for the detection of mutations and diseases [34,83]. Targeted NGS is significantly more cost-effective and efficient than WGS since it requires less sequencing and less time for analysis. Targeted NGS involves higher precision and accuracy and can simultaneously identify common and rare genetic variations. It has become a valuable routine technique in both research and clinical settings due to its high accuracy and lower cost [34,83].

Shah et al. demonstrated the feasibility of a ctDNA assay tailored for pediatric sarcomas, utilizing CAPP-Seq to detect translocations and CNAs with high sensitivity. In a cohort of 17 patients, translocations were identified in 81% of cases, with ctDNA levels correlating with metastatic status and treatment response. Notably, rising ctDNA levels preceded clinical relapse, suggesting its potential as an early biomarker for disease progression [36].

Demoret et al. evaluated the concordance between ctDNA profiles and tumor tissue sequencing in 24 patients with STSs. The study found that ctDNA detection was feasible in 77% of cases, with a moderate overall concordance rate of 51% for shared alterations and higher agreement observed for SNVs compared to other mutation types. More than 50% of samples showed TF < 1%, limiting the reliability of the test [46]. McConnell and Gazdova et al. designed a sarcoma-specific NGS panel, a form of targeted NGS used to detect translocations and CNVs and their corresponding matched cfDNA in tissue samples from 12 patients with STS [45]. These STSs included ARMS, ES, synovial sarcoma, and others. The researchers found that the sarcoma-specific NGS panel identified genetic and structural variants in 11/12 of the tissue samples and 6/12 of the plasma samples. The structural variants were detected in cfDNA at a >0.2% variant allele frequency. Based on these findings, targeted NGS in ctDNA has great clinical potential in monitoring sarcomas. Since the sample size was small, experiments need to be validated in larger cohorts to evaluate the efficacy of targeted NGS in stratifying sarcoma treatment response and health outcomes [45].

Gastrointestinal stromal tumor (GIST) is the most prevalent sarcoma of the GI tract [84]. Studies have shown that GIST-specific targeted NGS can detect ctDNA levels and establish the potential for treatment stratification and monitoring. Arshad and Roberts et al. utilized NGS to detect mutations in blood samples from 243 GIST patients [84]. Of those with known pathogenic mutations, KIT was most common (56%), followed by NF (7%), PDGFRA (6%), PI3KCA (6%), KRAS (5%), and others (6%). The majority of the tumors had an actionable KIT or PDGFRA mutation. When comparing ctDNA detection with tissue NGS, a positive predictive value (PPV) of 100% was reported. Based on these findings, ctDNA provides a rapid, noninvasive analysis of current mutations for patients with metastatic GIST [84]. ctDNA-based testing can help define optimal therapy based on resistance mutations.

Heinrich and Jones et al. used a GIST-specific NGS panel to retrospectively investigate whether 453 patients with advanced GIST and resistance to imatinib chemotherapy may better respond to ripretinib or sunitinib treatment [47]. Through their analysis, they confirmed that ctDNA sequencing identified a molecular subset of patients who may benefit from second-line treatment with ripretinib. These findings suggest how ctDNA sequencing may improve the prediction of drug therapy efficacy in advanced GIST patients [47].

Johansson and Berndsen et al. also developed a GIST-specific NGS panel. They monitored tumor-specific and tyrosine-kinase inhibitor (TKI) resistance mutations in cfDNA and applied this approach to patients undergoing surgical treatment [48]. Their GIST-specific NGS technique allowed for the detection of tumor-specific mutations and TKI resistance mutations with a mutant allele frequency of <0.1%. They reported that high-risk patients expressed higher ctDNA levels, and patients with detectable ctDNA expressed statistically significant higher tumor cell proliferation rates and tumor sizes. All patients who exhibited ctDNA-positive levels pre-surgery became ctDNA-negative post-surgery [48]. These valuable findings highlight how ctDNA can be utilized as a clinical biomarker in predicting treatment efficacy and aiding in decision-making for high-risk GIST patients.

Interestingly, targeted NGS has been shown to achieve more than just structural variant detection in sarcomas. Several studies have used targeted NGS to correlate ctDNA levels with sarcoma tumor burden and clinical outcome. When applied to clinical routine monitoring, it is an extremely valuable treatment stratification tool.

A study by Eisenhardt and Brugger et al. designed a synovial sarcoma-specific NGS panel to determine if ctDNA quantification and detection can be used to understand local or distant tumor recurrence [52]. The researchers found a ctDNA breakpoint detection specificity of 100% and sensitivity of 40%, which are unprecedented findings. Increased mutations increased assay sensitivity. The extracted ctDNA levels statistically significantly correlated with clinical outcomes and tumor burden. Following the resection of synovial sarcoma tumors, the authors observed declines in ctDNA concentrations. Upon the detection of metastases, ctDNA levels increased, highlighting how ctDNA correlated with clinical outcomes and detected recurrence. These findings show great promise for the routine clinical detection of tumor recurrence and tumor heterogeneity monitoring. Additionally, ctDNA levels show potential as a valuable tool for risk stratification, especially in guiding chemotherapy decisions. Eisenhardt and Brugger et al.’s assay is highly adaptable to other translocation-driven tumors as proven by applications to myxolipoid sarcoma [52]. These insights require further exploration in prospective clinical trials.

Very similarly, a study by Braig and Runkel et al. performed targeted NGS to analyze ctDNA concentrations in three STS tumors: myxofibrosarcoma, LMS, and undifferentiated pleomorphic sarcoma [49]. Their objective was to correlate ctDNA levels with tumor burden. Braig and Runkel et al. demonstrated that in cases of full remission, ctDNA was undetectable. Patients with later-stage recurrence showed low levels of ctDNA during clinical remission, indicating minimal residual disease. In active disease, ctDNA levels were statistically significantly elevated. Braig and Runkel et al. established an observable direct response to treatment as ctDNA levels declined following tumor resections, radiotherapy, and chemotherapy. The researchers concluded that ctDNA quantification alone is not sufficient to monitor tumor activity but provides significant information to aid in decision-making, the early detection of recurrence or metastases, and treatment responses for STSs [49].

Another study was able to correlate clinical tumor burden with ctDNA levels in STS. Zhou and Bui et al. utilized targeted NGS to detect ctDNA levels in 148 plasma samples of LMS tumors [50]. The samples were stratified into four sub-cohorts based on ctDNA kinetics and were tracked longitudinally with disease progression and recurrence. They found that the group with the best clinical outcome had the lowest ctDNA levels. Zhou and Bui’s results suggest that ctDNA elevation may indicate progressive disease, allowing for closer monitoring of patients for clinical intervention [50]. ctDNA analysis holds the potential to be a valid tumor biomarker for disease recurrence prediction and decision-making guidance in patients with LMS. With validation, longitudinal ctDNA monitoring can be applied to the clinical setting to inform treatment decisions and predict disease recurrence, improving survival outcomes in patients with LMS.

A recent study by Bui and Nemat-Gorgani et al. monitored ctDNA in 30 patients with advanced STS receiving a combination of ipilimumab, nivolumab, and cryoablation. The study included patients with UPS (37%), LMS (30%), synovial sarcoma (10%), MPNST (10%), and other subtypes (13%). ctDNA was analyzed using ULP-WGS to assess CNAs and bespoke CAPP-Seq ctDNA assays. ctDNA was detectable in 96% of patients, with higher pretreatment levels correlating with worse response and shorter PFS and OS. Post-cryoablation, ctDNA levels increased in 90% of patients but declined in responders. ORR was 4% by RECIST and 11% by irRECIST, with a median PFS of 2.7 months and OS of 12.0 months. ctDNA dynamics mirrored radiographic disease progression, with rising levels preceding clinical progression in some cases. Patients with a ≥50% reduction in ctDNA levels after treatment had improved PFS and OS [42].

Similarly, Eisenhardt and Schmid et al. developed a targeted NGS approach to detect ctDNA in myxoid liposarcoma and correlated ctDNA levels with tumor burden and health outcomes [51]. They reported that ctDNA levels decreased after tumor resection and increased when metastatic disease was detected. They found declining ctDNA levels with the initiation of radiotherapy and chemotherapy. Similarly to previously mentioned studies, they concluded that ctDNA quantification through targeted NGS could help predict tumor recurrence and monitor tumor heterogeneity and treatment response in metastatic disease with minimal invasiveness and at an affordable cost. Their assay can be easily adapted to other translocation-driven tumors, such as synovial sarcomas. Interestingly, Eisenhardt and Schmid also suggested that high ctDNA levels in myxoid liposarcoma patients with lung metastases may enable the detection of recurrence earlier via LB compared to imaging-based approaches [51].

Targeted NGS holds tremendous promise in applying ctDNA detection to sarcoma risk prediction, disease burden monitoring, and treatment response stratification. As previously shown, targeted NGS is highly capable of detecting CNVs and translocations in sarcoma samples. Additionally, studies have demonstrated a correlation between ctDNA levels, tumor burden, and disease recurrence using targeted NGS. ctDNA analysis through targeted NGS was performed for several sarcoma types, with high sensitivity and efficacy demonstrated across all types of sarcoma tumors.

#### 2.2.5. Summary of ctDNA Applications in Sarcoma and Future Directions

ctDNA monitoring has emerged as a promising noninvasive biomarker with significant clinical applications in the management of STS, offering advantages in diagnosis, disease monitoring, and treatment stratification. In the diagnostic setting, ctDNA enables the detection of characteristic structural variants, translocations, CNA, and epigenetic alterations, providing molecular insights that complement traditional tissue biopsies while overcoming challenges posed by tumor heterogeneity and limited biopsy accessibility. For disease monitoring, ctDNA levels correlate strongly with tumor burden, therapeutic response, and recurrence risk across multiple STS subtypes. Studies suggest that ctDNA may offer superior sensitivity over imaging in identifying minimal residual disease and detecting relapses at earlier time points, potentially allowing for preemptive therapeutic interventions; however, large-scale clinical validation remains limited. Advanced detection platforms, including ddPCR, targeted NGS, LP-WGS, and ULP-WGS, have refined the precision and clinical utility of ctDNA analysis, enabling its integration into therapeutic or surveillance trials for longitudinal disease. Moreover, ctDNA holds promise in real-time treatment adaptation, facilitating the detection of emerging resistance mutations and guiding personalized therapeutic strategies.

However, several challenges limit its widespread clinical adoption. Sarcomas generally shed low levels of cfDNA, restricting ctDNA detection and limiting its utility as a biomarker. This limitation underscores the need for more sensitive, consistent, and cost-effective detection methods. Additionally, the heterogeneity of STS subtypes complicates ctDNA analysis as current techniques are not fully optimized for all histologies. While some sarcomas with recurrent genomic alterations (e.g., GIST with KIT/PDGFRA mutations) exhibit higher ctDNA detectability, most (e.g., low-grade sarcomas, synovial sarcoma, and chondrosarcoma) remain challenging to detect due to low shedding and technical constraints in detecting complex structural variants. Future advancements in ultra-sensitive sequencing techniques, such as error-corrected NGS and methylation-based approaches, may enhance ctDNA detection across these subtypes.

Beyond current applications, ctDNA holds untapped potential in sarcoma management. Ongoing research is being conducted to investigate its role in identifying minimal residual disease post-resection, distinguishing post-treatment fibrosis from viable tumor recurrence, and assessing tumor mutational burden or HLA loss of heterozygosity, which may predict immunotherapy response in select sarcoma subtypes. Additionally, ctDNA detection may be adapted as a screening tool for high-risk populations, such as individuals with hereditary cancer syndromes like Li–Fraumeni syndrome. However, the lack of assay standardization and variability in ctDNA methodologies—as seen in studies comparing LP-WGS and methylation-specific ddPCR in osteosarcoma—remains a key barrier to clinical translation.

Ongoing clinical trials are aiming to validate ctDNA as a prognostic and predictive biomarker in sarcoma treatment. Several trials are investigating its association with prognosis, treatment response, and disease monitoring. NCT04925089 and NCT05653388 are evaluating ctDNA as a biomarker of chemotherapy response in leiomyosarcoma, while NCT06068075 is assessing its prognostic value in post-treatment Ewing sarcoma and osteosarcoma recurrence. Additionally, NCT03896620 is exploring ctDNA as a predictive tool for treatment response in metastatic and soft tissue sarcomas.

Future research should focus on prospective validation in clinical trials, the standardization of assay methodologies, and integration into risk-adapted treatment algorithms. Additionally, current ctDNA detection platforms require further optimization to improve sensitivity, specificity, and reliability to address the intrinsic challenges of STS as a low ctDNA-shedding malignancy, particularly in low-burden disease states and heterogeneous sarcoma subtypes, where ctDNA levels may be near the detection threshold. Refining pre-analytical processing, enhancing bioinformatic pipelines, and developing multimodal approaches that integrate ctDNA with complementary biomarkers, such as circulating RNA and proteomic signatures, may further improve clinical applicability. Ultimately, these advancements will be crucial in positioning ctDNA as a cornerstone biomarker in the precision management of STS.

## 3. Conclusions

STSs are rare and heterogeneous cancers with complex genetic profiles, presenting significant challenges in both diagnosis and treatment. Despite advances in therapy, metastatic STS remains difficult to manage, underscoring the urgent need for novel biomarkers and more effective therapeutic strategies. One promising approach is the use of ctDNA as a noninvasive biomarker for disease monitoring, prognosis, and treatment response. LB technologies, particularly ctDNA detection, have shown potential in providing insights into sarcoma genomics, offering a way to detect oncogenic mutations, monitor tumor burden, and assess therapy outcomes without the need for invasive biopsies.

Recent advances in ctDNA detection techniques, including PCR-based methods, NGS, and ddPCR, have improved sensitivity and accuracy in identifying sarcoma-specific mutations, structural variants, and chromosomal aberrations. These technologies have been applied across a range of sarcoma subtypes, from ES to OS, with promising results indicating that ctDNA levels correlate with tumor burden, treatment response, and disease recurrence. For example, ctDNA levels have been shown to fluctuate with tumor size and progression, making ctDNA a valuable tool for real-time monitoring. Furthermore, technologies such as LP-WGS and ULP-WGS have demonstrated their ability to detect CNAs and mutations in sarcoma patients, offering significant potential for early detection and risk stratification.

While the clinical application of ctDNA in sarcomas is still in its early stages, studies suggest that ctDNA analysis can help predict survival outcomes, detect minimal residual disease, and guide treatment decisions. For instance, the presence of ctDNA in plasma samples has been associated with poor prognosis in several sarcoma types, including RMS and LMS. Additionally, targeted NGS panels designed for specific sarcoma subtypes have shown high specificity and sensitivity, reinforcing the value of ctDNA in monitoring treatment efficacy and recurrence detection.

In conclusion, ctDNA presents a promising avenue for improving sarcoma management from diagnosis to treatment monitoring. As research continues to validate and refine these technologies, ctDNA could become an integral part of personalized treatment plans, helping clinicians make more informed decisions and potentially improving patient outcomes. Further large-scale studies and clinical trials are needed to establish ctDNA as a standard biomarker for sarcoma diagnosis and prognosis.
